# Loss of BID Delays FASL-Induced Cell Death of Mouse Neutrophils and Aggravates DSS-Induced Weight Loss

**DOI:** 10.3390/ijms19030684

**Published:** 2018-02-28

**Authors:** Simone Wicki, Ursina Gurzeler, Nadia Corazza, Vera Genitsch, Wendy Wei-Lynn Wong, Thomas Kaufmann

**Affiliations:** 1 Institute of Pharmacology, University of Bern, 3010 Bern, Switzerland; simone.wicki@pki.unibe.ch (S.W.); ursina.gurzeler@gymkirchenfeld.ch (U.G.); 2 Institute of Pathology, University of Bern, 3008 Bern, Switzerland; nadia.corazza@pathology.unibe.ch (N.C.); vera.genitsch@pathology.unibe.ch (V.G.); 3 Institute of Experimental Immunology, University of Zurich, 8057 Zurich, Switzerland; wong@immunology.uzh.ch

**Keywords:** neutrophil, FAS/CD95, BID, apoptosis, RIPK3, necroptosis, caspases, inflammation, colitis, mouse model

## Abstract

Neutrophils are key players in the early defense against invading pathogens. Due to their potent effector functions, programmed cell death of activated neutrophils has to be tightly controlled; however, its underlying mechanisms remain unclear. Fas ligand (FASL/CD95L) has been shown to induce neutrophil apoptosis, which is accelerated by the processing of the BH3-only protein BH3 interacting domain death agonist (BID) to trigger mitochondrial apoptotic events, and been attributed a regulatory role during viral and bacterial infections. Here, we show that, in accordance with previous works, mouse neutrophils underwent caspase-dependent apoptosis in response to FASL, and that this cell death was significantly delayed upon loss of BID. However, pan-caspase inhibition failed to protect mouse neutrophils from FASL-induced apoptosis and caused a switch to RIPK3-dependent necroptotic cell death. Intriguingly, such a switch was less evident in the absence of BID, particularly under inflammatory conditions. Delayed neutrophil apoptosis has been implicated in several auto-inflammatory diseases, including inflammatory bowel disease. We show that neutrophil and macrophage driven acute dextran sulfate sodium (DSS) induced colitis was slightly more aggravated in BID-deficient mice, based on significantly increased weight loss compared to wild-type controls. Taken together, our data support a central role for FASL > FAS and BID in mouse neutrophil cell death and further underline the anti-inflammatory role of BID.

## 1. Introduction

Neutrophils constitute the most abundant leukocytes in human peripheral blood and represent the first line defense against invading pathogens such as bacteria, fungi and certain viruses [1,2]. In the absence of infections, neutrophils are rapidly turned over by spontaneous apoptosis with an estimated lifespan of no more than a few days [3,4]. Importantly, bacterial components such as LPS and pro-inflammatory cytokines such as granulocyte-macrophage colony-stimulating factor (GM-CSF) prolong survival of neutrophils, which is crucial for an efficient antimicrobial response, by induction of anti-apoptotic genes, including MCL-1, BFL-1/BCL-2-A1 or X-linked inhibitor of apoptosis (XIAP) [5,6,7,8]. At a delayed stage, the same stimuli also induce pro-apoptotic BIM, thereby guaranteeing the timely removal of activated neutrophils [7,9]. Extended survival of activated neutrophils is known to cause collateral tissue damage and chronic inflammation. Dysregulated neutrophil death is implicated in several diseases such as inflammatory bowel disease (IBD), rheumatoid arthritis, cystic fibrosis and chronic obstructive pulmonary disease [3,10]. Therefore, neutrophil cell death must be tightly controlled, which is achieved by engagement of death receptors, phagocytosis of pathogens or decrease of survival factors [11]. However, the exact mechanisms of neutrophil cell death are not fully understood. Recently, it was demonstrated that neutrophils are killed by engagement of the death receptor FAS/CD95 during viral and bacterial infections [12,13]. FAS is constitutively expressed on most cell types while its ligand, Fas ligand (FASL), is mainly expressed on activated T cells, NK and NKT cells [14,15]. The FASL > FAS system is crucial in maintaining cellular homeostasis of the immune system and in preventing development of autoimmunity and cancer [16]. Intriguingly, FASL > FAS can not only induce apoptosis but also necroptosis, and is a potent inducer of NF-κB and chemokine and cytokine production depending on the cellular context as well as on the form (i.e., soluble vs. membrane-bound) of Fas ligand [17,18,19]. Soluble FASL induces cytokine production and survival whereas only the membrane-bound form can elicit cell death [20]. Binding of the membrane-bound FASL to its receptor triggers clustering of the receptors and formation of the death-inducing signaling complex, DISC, which constitutes of FADD and procaspase-8 [21]. Formation of the DISC facilitates the proteolytic trans auto-activation of pro-caspase-8 into its fully active form, which is then released from the DISC into the cytosol. As a result, caspase-8 directly activates effector procaspase-3 and -7. Caspase-8 also processes the BH3-only protein BH3 interacting domain death agonist (BID) into its active form tBID (p15) and thereby amplifies the caspase activation cascade by engaging the intrinsic, also called mitochondrial, apoptotic pathway [22,23].

According to their response to FAS stimulation, cells are divided in type I and type II like cells. In type I cells, induction of the extrinsic pathway is sufficient to kill the cells whereas in type II cells, for example in hepatocytes or pancreatic β cells, tBID-mediated crosstalk to the intrinsic pathway is required for efficient FAS-induced cell death [24,25]. There is some evidence that loss of BID delays FASL-induced neutrophil death, suggesting that neutrophils are type II-like cells, whereas, interestingly, TNFα > TNF-R1-induced neutrophil cell death does not depend on BID [26,27].

Neutrophils are crucial players in the maintenance of intestinal homeostasis but their accumulation in the intestine results in mucosal injury and thereby promotes development of IBD [28]. Dextran sulfate sodium (DSS)-induced colitis depends on neutrophils and macrophages and is a suitable model to mimic clinical manifestations of IBD observed in humans. DSS affects epithelial cells in the colon and causes disruption of the epithelial barrier. As a consequence DSS and other large molecules may penetrate the colon mucosa and trigger an inflammatory response involving neutrophils [29]. Programmed cell death of activated neutrophils (and subsequent elimination by macrophages), which is delayed in patients suffering from IBD, is absolutely critical for the resolution of inflammation [28]. This is also supported from studies in mice in which depletion of neutrophils diminished the severity of DSS-induced colitis [30]. Furthermore, enhanced expression of FASL could be detected in inflamed intestine and therefore, FASL may promote resolution of experimental colitis [31]. In line with this observation, mice expressing a signaling deficient mutant of FAS (*lpr^cg^* mice) are more susceptible to DSS-induced colitis [32].

Here, we examined the regulation of FASL > FAS induced cell death in mouse neutrophils and the involvement of BID in these processes. We observed that loss of BID did not abolish but significantly delayed FASL-mediated effector caspase activation and subsequent cell death, strongly supporting the notion that neutrophils are type II-like cells. Even though neutrophils underwent caspase-mediated apoptosis in response to FASL, pan-inhibition of apoptotic caspases could not fully restore viability. Additional enzymatic inhibition of RIPK1 or genetic ablation of RIPK3 was required to protect neutrophils from FASL-induced killing suggesting that a switch to necroptosis occurs when caspases are blocked. To support our in vitro findings, we compared WT and *Bid*^−/−^ mice in an acute DSS-colitis model. We observed that loss of BID worsened the severity of colitis, based on the overall weight loss, and delayed resolution probably due to accumulation of activated neutrophils in the colon. Overall, our results implicate that BID importantly regulates neutrophil death in the context of FASL > FAS and DSS colitis and that through this anti-inflammatory function BID may counteract development of inflammatory diseases such as IBD.

## 2. Results

### 2.1. Fas Ligand (FASL) Induces Neutrophil Death, Which Is Delayed in Absence of BH3 Interacting Domain Death Agonist (BID)

**Figure 1:**
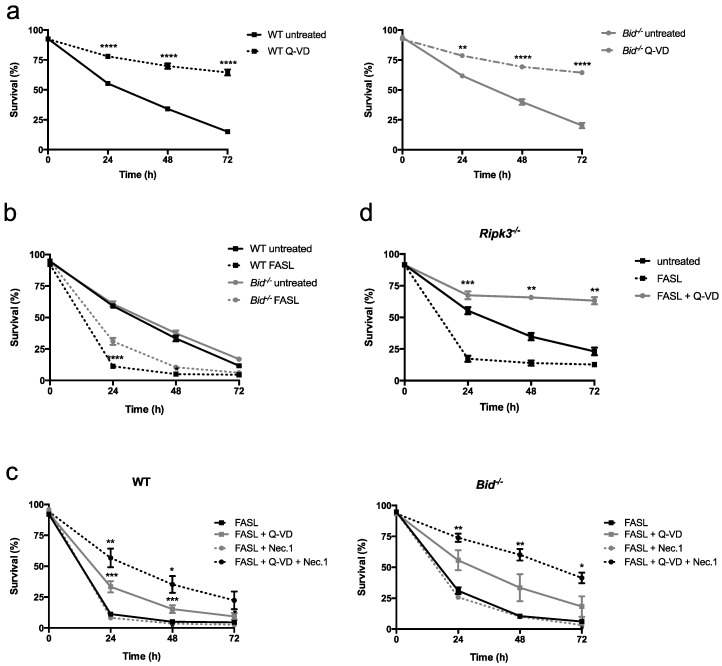
Mouse neutrophils undergo cell death in response to FASL, which is delayed by loss of BID. (**a**) Assessment of viability of WT and *Bid*^−/−^ neutrophils left untreated (spontaneous apoptosis) or treated with the pan-caspase inhibitor Q-VD-OPh (20 μM) for indicated time points. *n* ≥ 6, mean ± SEM. (**b**) Assessment of viability of WT and *Bid*^−/−^ neutrophils upon treatment with FASL (100 ng/mL) for indicated time points. *n* ≥ 3, mean ± SEM. (**c**) WT and *Bid*^−/−^ neutrophils were pre-treated with Q-VD-OPh (20 μM) and/or Nec.1 (20 μM) for 30 min followed by treatment with FASL (100 ng/mL) for indicated time points. Viability was assessed by flow cytometry. *n* ≥ 4, mean ± SEM. (**d**) *Ripk3*^−/−^ neutrophils were pre-treated with Q-VD-OPh (20 μM) for 30 min and subsequently stimulated with FASL (100 ng/mL) for indicated time points. Viability was assessed by flow cytometry using GFP-AnnexinV/PI exclusion. *n* = 3, mean ± SEM. All experiments were performed with primary bone marrow-derived neutrophils. *p* < 0.05 (*), *p* < 0.01 (**), *p* < 0.005 (***) and *p* < 0.001 (****).

Upon in vitro culturing in the absence of specific cytokines, neutrophils undergo spontaneous apoptosis. As shown in Figure 1a, spontaneous apoptosis of primary bone marrow-derived mouse neutrophils occurred by a classical caspase-dependent mechanism, as it was fully blocked by the pan-caspase inhibitor Q-VD-OPh. Wild-type (WT) and *Bid*^−/−^ neutrophils died with the same kinetics, indicating that BID does not contribute to spontaneous neutrophil apoptosis (Figure 1a,b). We next tested the effect of crosslinked FASL on the viability of WT and *Bid*^−/−^ neutrophils over time. Primary neutrophils were isolated from the bone marrow of mice or, alternatively, neutrophils were differentiated in vitro using conditional Hoxb8, which allows working with many cells [33,34,35]. Primary as well as in vitro differentiated WT neutrophils were highly sensitive to FASL-induced cell death, whereas cell death was significantly delayed upon loss of BID (Figure 1b and Figure S1a). Of note, *Bim*^−/−^ primary neutrophils died with the same kinetics in response to FASL as WT controls (Figure S2). Interestingly, pan-inhibition of apoptotic caspases by Q-VD-OPh had only a partial protective effect from FASL in WT cells, whereas the viability of *Bid*^−/−^ neutrophils was again higher and comparable to untreated controls (Figure 1c and Figure S1b). Additional inhibition of the enzymatic activity of RIPK1 by necrostatin-1 was required to further increase viability in FASL-treated cells (Figure 1c and Figure S1b). However, necrostatin-1 alone had no protective effect (Figure 1c). Furthermore, genetic ablation of RIPK3 resulted in a full protection from FASL-induced killing by Q-VD-OPh, with significantly higher viabilities compared to untreated controls (spontaneous apoptosis) at later timepoints (Figure 1d and Figure S1c). Taken together, these data suggest that FASL-induced apoptosis may switch to RIPK1- and RIPK3-dependent cell death when caspases are blocked and that, interestingly, BID seems to contribute to such a switch.

### 2.2. Neutrophils Undergo Apoptosis in Response to FASL, Which Switches to Necroptosis Upon Inhibition of Caspases

**Figure 2:**
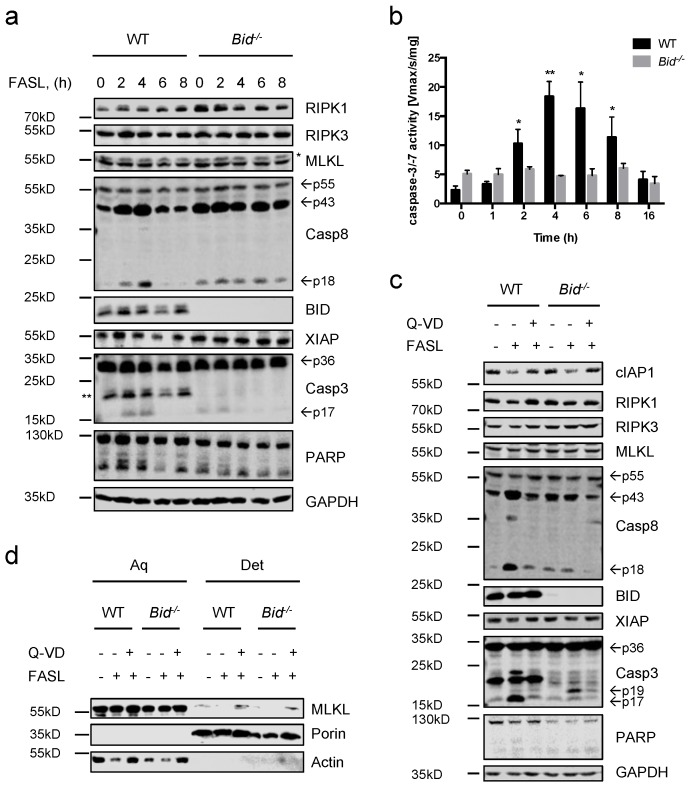
FASL induces apoptosis in neutrophils, which switches to necroptosis when caspases are inhibited. (**a**) WT and *Bid*^−/−^ neutrophils were treated with FASL (100 ng/mL) for 0–8 h. Lysates were assessed by immunoblotting. Presented immunoblots are representative of at least two independent experiments. * indicates non-specific bands; ** indicates bands derived from previous anti-BID immunoblotting. (**b**) WT and *Bid*^−/−^ neutrophils were stimulated with FASL (100 ng/mL) for indicated time points. Lysates were assayed for caspase-3/-7 activity by fluorogenic DEVDase assay. *n* ≥ 3, mean ± SEM. *p* < 0.05 (*), *p* < 0.01 (**). (**c**) WT and *Bid*^−/−^ neutrophils were pre-treated with Q-VD-OPh (20 μM) for 30 min and subsequently treated with FASL (100 ng/mL) for 6 h. Lysates were subjected to immunoblotting. Presented immunoblots are representative of at least two independent experiments. (**d**) WT and *Bid*^−/−^ neutrophils were pre-stimulated with Q-VD-OPh (20 μM) for 30 min followed by administration of FASL (100 ng/mL) for 16 h. Fractionation by phase separation was performed and lysates were subjected to immunoblotting. Presented immunoblots are representative of at least two independent experiments. All experiments were performed with in vitro differentiated neutrophils.

To further investigate the type of FASL-induced cell death, we analyzed protein lysates prepared from FASL-stimulated neutrophils at various time points by immunoblotting. As shown in Figure 2a,c, stimulation of FASL caused rapid processing of initiator caspase-8 into its fully active p18 fragment in WT cells. Cleavage of caspase-8 concurred with processing of procaspase-3 into its active form as well as cleavage of the caspase-3 substrate PARP. Additionally, enzymatic activation of caspase-3/-7 was confirmed by fluorogenic DEVDase assay. Caspase-3/-7 activity was observed within a few hours and peaked after 4 h. However, in absence of BID, much lower caspase-3/-7 processing and enzymatic activity levels could be measured, consistent with the observed delay in cell death in Figure 1 (Figure 2a–c). Moreover, a slight decrease in XIAP protein expression was detected over time in WT neutrophils, which was not apparent in the absence of BID (Figure 2a). Interestingly, cIAP1 protein levels strongly decreased in response to FASL in both WT and *Bid*^−/−^ neutrophils through a caspase-dependent mechanism (Figure 2c). Due to the findings that inhibition of caspases could not fully restore viability upon FASL treatment, translocation of MLKL from an aqueous fraction to a detergent (integral membrane enriched) fraction, which is considered a hallmark of necroptosis [36], was assessed. MLKL was found only in the aqueous fraction in response to FASL alone while upon blockage of caspases MLKL was also found in the detergent fraction in both WT and *Bid*^−/−^ neutrophils, suggesting translocation of MLKL to membranes (Figure 2d). Taken together, our data show that, in response to FASL, mouse neutrophils undergo caspase-dependent apoptosis, which is accelerated by BID. However, in the absence of caspase activity cell death switches from apoptosis to necroptosis with translocation of MLKL to membranes. 

### 2.3. FASL Efficiently kills Granulocyte-Macrophage Colony-Stimulating Factor (GM-CSF)- and LPS-Primed Neutrophils

**Figure 3:**
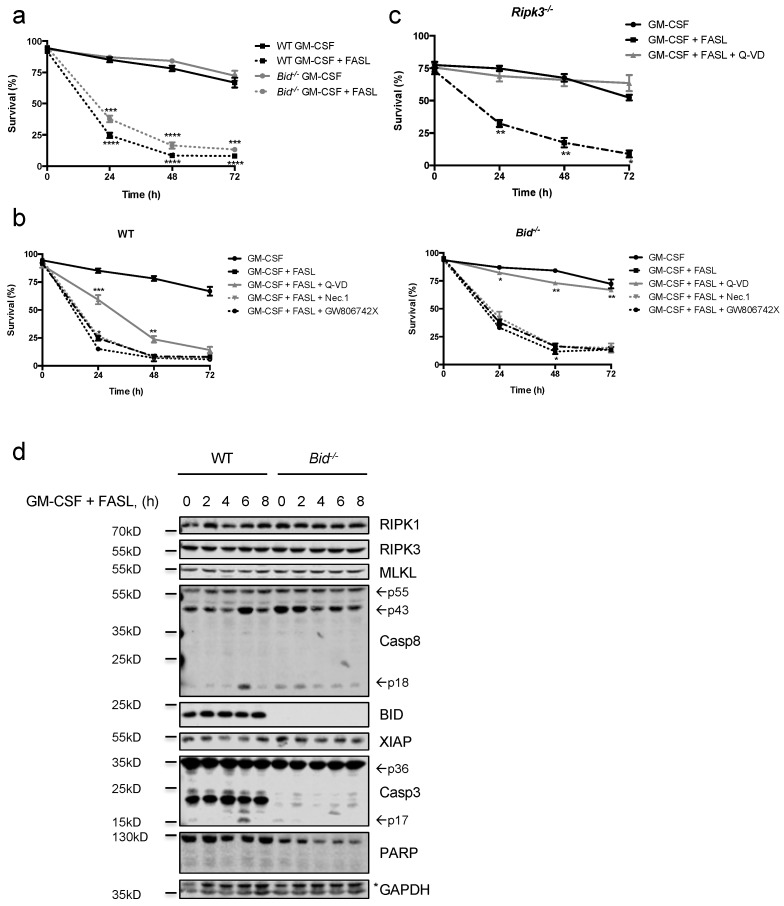
GM-CSF-primed neutrophils remain sensitive to FASL killing. (**a**) Primary WT and *Bid*^−/−^ neutrophils were primed with GM-CSF (1 ng/mL) for 30 min prior to stimulation with FASL (100 ng/mL) for indicated time points. Viability was assessed by flow cytometry. *n* ≥ 3, mean ± SEM. (**b**) Primary WT and *Bid*^−/−^ neutrophils were primed with GM-CSF (1 ng/mL) for 30 min and pre-treated with either Q-VD-OPh (20 μM), Nec.1 (20 μM) or the mouse MLKL inhibitor GW806742X (1 μM) for 30 min followed by stimulation with FASL (100 ng/mL) for indicated time points. Viability was assessed by flow cytometry. *n* ≥ 3, mean ± SEM. (**c**) In vitro differentiated *Ripk3*^−/−^ neutrophils were primed with GM-CSF (1 ng/mL) for 30 min prior to stimulation with FASL (100 ng/mL) with or without Q-VD-OPh (20 μM) for indicated time points. Viability was assessed by flow cytometry. *n* ≥ 3, mean ± SEM. (**a**–**c**): *p* < 0.05 (*), *p* < 0.01 (**), *p* < 0.005 (***) and *p* < 0.001 (****). (**d**) In vitro differentiated WT and *Bid*^−/−^ neutrophils were primed with GM-CSF (1 ng/mL) for 30 min and subsequently treated with FASL (100 ng/mL) for 0–8 h. Lysates were assessed by immunoblotting. Presented immunoblots are representative of at least two independent experiments.

We then examined the effect of FASL on primed neutrophils, thus mimicking the conditions encountered in an inflammatory environment. The pro-inflammatory cytokine GM-CSF as well as bacterial endotoxin LPS are known to prime neutrophils and to prolong their survival [5,34]. We have recently shown that unprimed neutrophils are sensitive to TNFα-induced apoptosis, whereas this effect is fully lost upon priming with GM-CSF [34]. In contrast, GM-CSF-primed primary as well as in vitro differentiated WT neutrophils remained highly sensitive to FASL-induced cell death, and loss of BID slightly but significantly delayed death kinetics (Figure 3a and Figure S3). GM-CSF-primed WT neutrophils could only be partly protected by pan-caspase inhibition whereas neither inhibition of RIPK1 nor MLKL had a protective effect on their own (Figure 3b). Intriguingly, GM-CSF primed *Bid*^−/−^ neutrophils could be fully protected with the pan-caspase inhibitor Q-VD-OPh, indicating that, under primed conditions, BID importantly contributes to the switch from apoptosis to caspase-independent cell death (Figure 3b). This caspase-independent cell death is likely necroptosis, as GM-CSF primed *Ripk3*^−/−^ neutrophils remained sensitive to FASL but this cell death was now fully blockable by pan-caspase inhibition (Figure 3c). In contrast to unprimed neutrophils, primed neutrophils died with slower kinetics. Cleaved caspase-8 and -3 could be detected at later time points compared to unprimed neutrophils, which correlated with delayed cell death. Again, a decrease over time of XIAP in WT, but not *Bid*^−/−^, neutrophils could be observed (Figure 3d).

**Figure 4:**
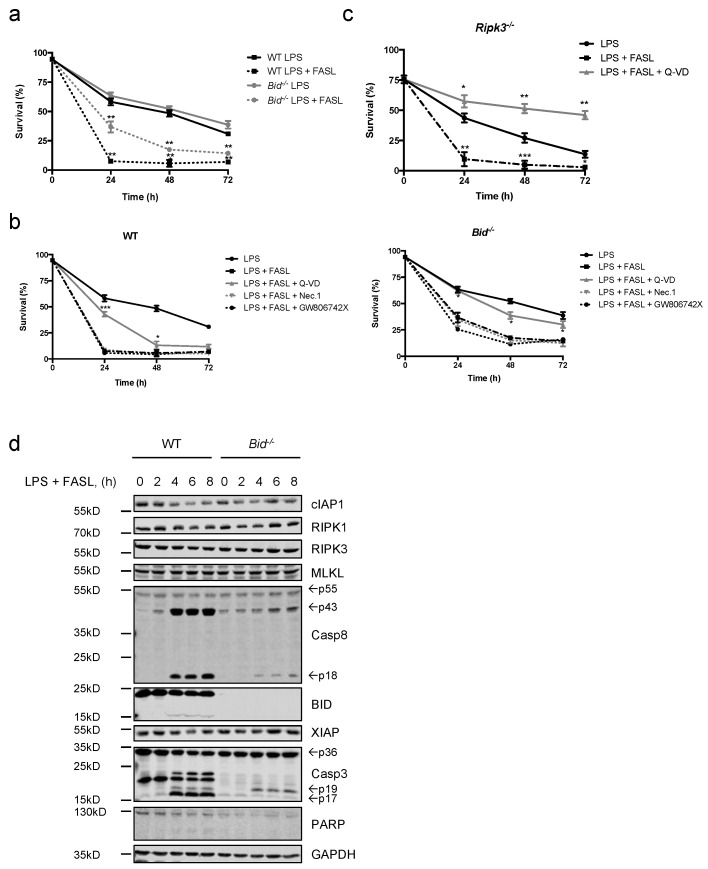
LPS-primed neutrophils remain sensitive to FASL killing. (**a**) Primary WT and *Bid*^−/−^ neutrophils were primed with LPS (10 ng/mL) for 30 min prior to stimulation with FASL (100 ng/mL) for indicated time points. Viability was assessed by flow cytometry. *n* ≥ 3, mean ± SEM. (**b**) Primary WT and *Bid*^−/−^ neutrophils were primed with LPS (10 ng/mL) for 30 min, pre-treated with either Q-VD-OPh (20 μM), Nec.1 (20 μM) or the mouse MLKL inhibitor GW806742X (1 μM) for 30 min followed by stimulation with FASL (100 ng/mL) for indicated time points. Viability was assessed by flow cytometry. *n* = 3, mean ± SEM. (**c**) In vitro differentiated *Ripk3*^−/−^ neutrophils were primed with LPS (10 ng/mL) for 30 min prior to stimulation with FASL (100 ng/mL) with or without Q-VD-OPh (20 μM) for indicated time points. Viability was assessed by flow cytometry. *n* ≥ 3, mean ± SEM. (**a**–**c**): *p* < 0.05 (*), *p* < 0.01 (**) and *p* < 0.005 (***). (**d**) In vitro differentiated WT and *Bid*^−/−^ neutrophils were primed with LPS (10 ng/mL) prior to stimulation with FASL (100 ng/mL) for 0–8 h. Lysates were subjected to immunoblot. Presented immunoblots are representative of at least two independent experiments.

Similar to GM-CSF priming, LPS primed WT, *Bid*^−/−^ and *Ripk3*^−/−^ neutrophils were as sensitive as unprimed neutrophils to FASL-induced cell death, with *Bid*^−/−^ cells being more resistant than WT controls (Figure 4a–d and Figure S4). Furthermore, caspase inhibition resulted in a complete rescue of *Ripk3*^−/−^ neutrophils and viabilities were significantly higher in *Bid*^−/−^ neutrophils compared to WT controls (Figure 4b,c). Taken together, GM-CSF- or LPS-primed neutrophils remain highly sensitive to FASL-induced cell death and retain their type II characteristics. Furthermore, when caspases are blocked upon FASL stimulation, caspase-independent and RIPK3-dependent cell death is enabled by a mechanism facilitated by BID. 

### 2.4. Dextran Sulfate Sodium (DSS)-Induced Weight Loss in Mice Is Aggravated in the Absence of BID

**Figure 5:**
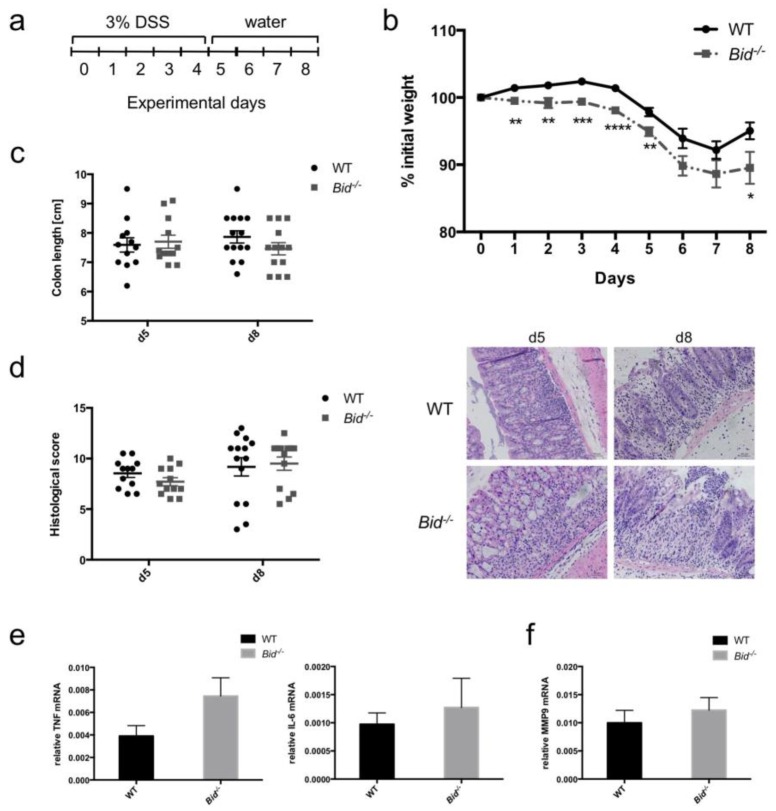
DSS-induced colitis is mildly exacerbated upon loss of BID. Colitis induced by 3% DSS in WT and *Bid*^−/−^ mice (*n* = 12–14 animals per time point and group). (**a**) Scheme of the experimental set up. (**b**) Time course of weight loss (*p* < 0.05 (*), *p* < 0.01 (**), *p* < 0.005 (***) and *p* < 0.001 (****)) and (**c**) colon length after Day 5 and Day 8 of treatment. (**d**) Histology of colon tissues stained with H&E after Day 5 and Day 8 of treatment and analysis of severity of colitis by histological scoring. Presented images are representative. (**e**) Relative *Tnfα* and *Il-6* mRNA expression in colon tissues after five days of treatment. *Gapdh* was used as reference gene. (**f**) Relative *Mmp9* mRNA expression in colon tissues after five days of treatment. *Gapdh* was used as reference gene.

To investigate the role of BID in development and resolution of acute colitis mediated predominantly by neutrophils and macrophages, we added 3% DSS to the drinking water for five days (Figure 5a). There was no difference in colon lengths between untreated WT and *Bid*^−/−^ mice (WT: 8.54 ± 0.24 cm, *Bid*^−/−^: 8.44 ± 0.28 cm; means ± SEM, *n* = 5 per genotype). Both WT and *Bid*^−/−^ mice developed colitis over time observed by constant weight loss and shortening of their colons (Figure 5b,c). As soon as DSS-containing water was replaced by normal water at Day 5 mice started to recover and gained weight again from Day 7 onwards. However, the weight loss in *Bid*^−/−^ mice was significantly more pronounced compared to WT mice and recovery phase was delayed (Figure 5b). DSS treated *Bid*^−/−^ mice showed a trend to shortened colons—consistent with increased inflammation—at the endpoint of eight days compared to WT mice, but this did not reach statistical significance (Figure 5c). Histopathological scoring from colon samples of *Bid*^−/−^ as well as WT mice displayed minor differences (Figure 5d). Slightly higher *Tnfα* and *Il-6* mRNA levels could be quantified in colon pieces from *Bid*^−/−^ mice after five days of treatment compared to WT controls (Figure 5e). Furthermore, differences in number of neutrophils in inflamed colon pieces were investigated. The matrix metalloprotease MMP9 was used as indication for neutrophil numbers, as activated neutrophils are a major source of this protease. *Mmp9* mRNA was slightly higher in colons of *Bid*^−/−^ mice after five days of treatment, suggesting a higher neutrophil count in the colon (Figure 5f). 

## 3. Discussion

Neutrophils are crucial for the first line defense against invading pathogens. Their lifespan has to be tightly controlled, especially once they enter an activated state, and neutrophil cell death is achieved amongst others by engagement of the death receptor FAS, which is constitutively expressed on neutrophils [1,2,11]. FAS has been proposed to contribute to the resolution of inflammation by killing immune cells including neutrophils [12,13,16]. To date, it has been shown that neutrophils undergo caspase-mediated apoptosis, which is fully blockable with pan-caspase inhibitors in human neutrophils [10,37]. We confirmed that the spontaneous cell death observed upon culturing of mouse neutrophils in the absence of specific cytokines occurs by classical caspase-dependent apoptosis. We focused our study on FASL > FAS mediated signaling in mouse neutrophils and could demonstrate that mouse neutrophils are highly sensitive to FASL and likewise undergo caspase-dependent apoptosis. However, and in contrast to human neutrophils, we show that FASL-induced cell death in mouse neutrophils switches to RIPK3-dependent necroptosis under conditions of caspase inhibition (Figure 1 and Figure 2). These findings are somewhat surprising, since we recently published that TNFα > TNF-R1 induced apoptosis in mouse neutrophils is fully blockable using a pan-caspase inhibitor [34]. As a possible reason might be the observed decrease in cIAP1 (and in WT cells also XIAP) protein expression found upon FASL stimulation. It has been shown for TRAIL-induced apoptosis that cIAP1 and XIAP are degraded by caspase-8 and the latter also by caspase-9 [38]. Whether this holds also true for FASL-induced neutrophil apoptosis is not clear and needs further investigation. Furthermore, the question arises why human neutrophils can apparently be protected from FAS-induced cell death by blocking caspases. One possible explanation could be the relatively high expression levels of RIPK3, RIPK1 and MLKL proteins in mouse neutrophils, which make them more prone to undergo necroptosis (Figure 2, Figure 3 and Figure 4) [34]. In contrast, RIPK3 protein expression levels seem to vary in humans from donor to donor and can be very low based on immunoblotting (personal communication Dr. X Wang, University of Bern, Switzerland). 

In agreement with previous works [26,27], we show that loss of BID delays neutrophil apoptosis induced by FASL underlining that neutrophils are type II cells. This contribution of BID to FASL-induced killing was further increased when neutrophils were primed with GM-CSF or bacterial LPS, supporting the importance of BID in cell death regulation of neutrophils in an inflammatory environment. When such an inflammatory environment was mimicked by addition of GM-CSF or LPS prior to stimulation with FASL, the primed neutrophils remained highly sensitive to FASL-induced killing, in sharp contrast to earlier findings on TNFα induced neutrophil death (34). This finding is in agreement with a previous report [39] and supports the notion that FAS may have crucial roles in controlling neutrophil death in order to resolve inflammation (Figure 3 and Figure 4) [16]. Intriguingly, the switch from apoptosis to RIPK3-dependent necroptosis observed under caspase inhibiting conditions seems to be at least partially dependent on BID, as it was less obvious in unprimed *Bid*^−/−^ neutrophils and basically absent in GM-CSF- or LPS-primed *Bid*^−/−^ neutrophils (Figure 1c, Figure 3b and Figure 4b). These data suggest a connection from BID, and thus likely from BID-mediated mitochondrial apoptotic events, to induction of RIPK3-dependent necroptosis. Even though such a connection is poorly understood and needs further investigation, a recent report by Giampazolias and colleagues showed that mitochondrial outer membrane permeabilisation (MOMP) can engage necroptosis [40]. 

Prolonged survival of activated neutrophils causes tissue damage and is implicated in numerous diseases including IBD and rheumatoid arthritis [3]. We used the DSS-induced colitis model in mice to investigate the role of BID in development and resolution of colitis (Figure 5). Based on the weight loss during the experiment, *Bid*^−/−^ mice developed worse colitis with delayed resolution compared to WT mice. These findings are in line with our in vitro data showing loss of BID has a positive impact on survival of FASL-treated neutrophils. Prolonged survival of activated neutrophils would thereby result in aggravated and extended inflammation. Even though the observed effects were modest, and with the exception of the weight loss did not reach statistical significance, our data are supportive of an anti-inflammatory role of BID [41] and do not confirm a recently proposed pro-inflammatory role [42]. However, a limitation of our in vivo study is the use of a conventional *Bid*^−/−^ mouse strain that lacks BID expression in all cell types. Loss of BID could also have an impact in other cells, such as macrophages or colon epithelial cells. Generation of conditional transgenic mice, such as for example a neutrophil-specific *Bid*-deficient strain, would allow definitive conclusions about the specific role of BID in neutrophils in DSS-induced colitis.

In summary, we show that BID is an important regulator downstream of FAS in mouse neutrophils. Loss of BID significantly delays FASL-cell death and partially counteracts the switch from apoptosis to necroptosis when caspases are blocked. Additionally, and in contrast to TNF-R1 signaling [34], GM-CSF- as well as LPS-primed neutrophils remain sensitive to FASL-induced cell death which supports a role of the FASL > FAS system in the regulation of neutrophil cell death under inflammatory conditions.

## 4. Materials and Methods

### 4.1. Mice and Reagents

C57BL/6 mice were maintained under pathogen-free conditions in IVC cages. *Bid*^−/−^ [43] and *Ripk3*^−/−^ mice [44] have been previously described. All animals were approved by the animal experimentation review board of the canton of Bern (BE4/12, BE31/1 and BE12/14). 

RPMI-1640 AQmedia^TM^, propidium iodide (PI) and 4-hydroxytamoxifen (4-OHT) were from Sigma-Aldrich Chemie (Buchs, Switzerland). Penicillin/streptomycin and 2-mercaptoethanol (2-ME) were purchased from Life Technologies (Carlsbad, CA, USA). CHO/SCF (mm) conditioned medium (used as a source of mouse stem cell factor) was produced as previously described [45]. Recombinant His_6_-tagged GFP-Annexin V was purified as previously described [46]. Fetal calf serum (FCS, Sera Pro, ultra-low endotoxin) was from Pan Biotech (Aidenbach, Germany). Recombinant FLAG-FASL (human) and necrostatin-1 were purchased from Enzo LifeSciences AG (Lausen, Switzerland). Q-VD-OPh was from SM Biochemicals (Anaheimm, CA, USA). GW806742X was from Synkinase (Parkville, VIC, Australia). Ultrapure LPS (*E. coli* K12) was from Invivogen (San Diego, CA, USA). Recombinant mouse GM-CSF was purchased from Peprotech (Rocky Hill, NJ, USA). 

### 4.2. Isolation of Primary Neutrophils from Murine Bone Marrow

Cells were harvested from femoral bones. Primary neutrophils cells were isolated using rat anti-Gr-1 antibody (clone RB6-8C5, BioLegend, San Diego, CA, USA) by magnetic bead based cell sorting following the manufacturer’s instructions (BD IMag^TM^, BD Biosciences, San Jose, CA, USA). The purity was in general above 95% as assessed by morphology following staining with DiffQuik solution (Baxter, Deerfield, IL, USA). Neutrophils were cultivated in RPMI-1640 AQmedia complemented with 10% FCS, penicillin/streptomycin and 50 μM 2-ME. 

### 4.3. Generation and In Vitro Differentiation of Mouse Neutrophils

Conditional Hoxb8 immortalized neutrophil/macrophage committed myeloid progenitors, termed SCF-^cond^Hoxb8 cells, were generated from bone marrow of WT, *Bid*^−/−^ and *Ripk3*^−/−^ mice as previously described [35,45,47]. Cells were cultivated in RPMI-1640 AQmedia complemented with 10% FCS, penicillin/streptomycin, 0.1 μM 4-OHT and 5% CHO/mmSCF-conditioned medium. Upon removal of 4-OHT, cells differentiated into mature neutrophils within 5 days. To confirm successful differentiation the following surface markers profile was used: Gr-1(Ly-6C/Ly-6G)^hi^ CD11b^+^ CD117(c-kit)^neg^, using the following antibodies from BioLegend (San Diego, CA, USA): rat anti-CD11b (clone M1/70), rat anti-Gr-1 (clone RB6-8C5), and rat anti-CD117 (c-kit, clone 2B8). All experiments were performed with mature neutrophils. 

### 4.4. Gel Electrophoresis and Immunoblotting

Cells were lysed in hot H8-Buffer (20 mM Tris/HCL pH 7.5, 2 mM EGTA, 2 mM EDTA, 1% SDS, supplemented before us with 50 mM DTT). Proteins were denatured in 4× Lämmli Buffer (complemented with 50 mM DTT), separated on 7.5–12.5% denaturing SDS-PAGE gels and subsequently transferred to PDVF membrane (Immobilon-FL, 0.45 μM, Merck Millipore, Zug, Switzerland). Membranes were probed with primary antibodies including: mouse anti-RIPK1 (clone 610458), mouse anti-PARP (clone C2-10), mouse anti-actin (C4/actin) and mouse anti-XIAP (clone 28/hILP/XIAP) from BD BioSciences; rabbit polyclonal anti-RIPK3 (Pro-Sci, #2283); rat anti-MLKL (clone 3H1; kind gift from W. S. Alexander, Melbourne); rabbit polyclonal anti-procaspase-3 (#9662), anti-cleaved caspase-3 (#9661) and rabbit anti-cleaved-caspase-8 (clone D5B2) from Cell Signaling; rat anti-procaspase-8 (clone 1G12, kind gift from L. O’Reilly, Melbourne); mouse anti-GAPDH (clone 6C5, Merck Millipore); rat anti-BID (clones 2D1 and 8C3; kind gift from D. Huang, Melbourne); mouse anti-porin (clone 89-173/016) from Calbiochem. Infrared dye-conjugated secondary antibodies (LI-COR Biosciences, Bad Homburg, Germany) were used for quantitative immunoblotting. Alternatively, horseradish peroxidase-conjugated secondary antibodies (Jackson ImmunoResearch Europe Ltd., Newmarket SFK, UK) were used and signals were obtained by enhanced chemiluminescence (Luminata^TM^ Forte Western HRP substrate, Merck Millipore). Immunoblots were analyzed with the Odyssey^®^ Fc Dual-Mode Imaging System using the ImageStudio software 3.1.4 (LI-COR).

### 4.5. Assessment of Cell Death by Flow Cytometry

Neutrophils were stained with GFP-Annexin V diluted in FACS Buffer (150 mM NaCl, 4 mM KCl, 2.5 mM CaCl_2_, 1 mM MgSO_4_, 15 mM HEPES pH 7.2, 2% FCS and 10mM NaN_3_) for 20 min on ice in the dark. After a washing step, propidium iodide (2 μg/mL) was added to the cells. Viability was determined by flow cytometry and analyzed using WEASEL version 3.0.2 [48]. Cells negative for both GFP-Annexin V and propidium iodide were considered as viable.

### 4.6. Detection of Active Caspase-3 and -7 Activity

A fluorometric assay with DEVD-AMC as substrate was performed to measure enzymatic activity of caspase-3 and -7 (Bachem, Bubendorf, CH). First, 1 × 10^6^ neutrophils were lysed in 50 μL digitonin lysis buffer (20 mM HEPES pH 7.4, 100 mM sucrose, 25 mM MgCl_2_, 100 mM KCl, protease inhibitor cocktail (Roche Complete protease inhibitor cocktail plus 1 μg/mL pepstatin), 1 mM DTT, 0.025% digitonin). Protein concentrations were assessed by BCA protein assay according to the manufacturer’s instructions (Thermo Fisher Scientific, Waltham, MA, USA). Protein lysates (10–25 μg) were mixed with assay buffer (0.1 M HEPES pH 7.5, 10% sucrose, 0.1% CHAPS, 10 mM DTT) complemented with 500 µM DEVD-AMC (final concentration 50 μM). Enzymatic activity was quantified kinetically for one hour (1-min intervals) on a SpectraMax M2^e^ plate reader (Molecular Devices, San Jose, CA, US).

### 4.7. Fractionation by Phase Separation

Fractionation was adapted from [49] and performed as previously described [34].

### 4.8. DSS-Induced Colitis

Eight- to twelve-week-old WT and *Bid*^−/−^ female mice were mixed in IVC cages and given 3% DSS (reagent-grade DSS salt, molecular mass: 36–50 kD, MP Biomedicals, Illkirch-Graffenstaden, FR) in normal drinking water for 5 days. At Day 5, DSS-containing water was replaced by normal water. Animals were sacrificed after 5 or 8 days, respectively. Loss of body weight was monitored daily during the entire course of the experiment. After sacrificing the mice, colon lengths were measured. Colons were snap frozen or fixed in 4% paraformaldehyde for further analysis. 

### 4.9. Histology Scoring

Fixed tissues were embedded in paraffin, cut and stained with hematoxylin and eosin. Histopathological alterations were scored by a pathologist using a scoring system with the following parameters: cellular infiltration in the lamina propria of the large bowel (score from 0 to 3), crypt abscesses (score from 0 to 3), loss of goblet cells (score 0 to 3), epithelial erosion (score 0 to 1), hyperemia (score from 0 to 2), and thickness of mucosa (score from 0 to 3). The range of scores was from 0 (not affected) to 15 (most severe).

### 4.10. qPCR Analysis

Total RNA was isolated from 5 × 10^6^ neutrophils using the SV total RNA isolation system (Promega, Wallisellen, Switzerland) and subsequently reverse-transcribed using oligo d(T) primers and M-MLV reverse transcriptase (Promega) according to the manufacturer’s instructions. Quantitative RT-PCR (qPCR) analysis was performed using HOT FIREPol^®^ EvaGreen^®^ qPCR Mix Plus from Solis Biodyne (Tartu, Estonia) on a Real-Time PCR machine (iQ5, Bio-Rad Laboratories AG, Cressier, Switzerland). Primer sequences were the following: *mTnfα* (TNFα, amplicon 128 bp) Fw 5′-ATGAGAAGTTCCCAAATGGC, Rev 5′-CACTTGGTGGTTTGCTACGAC; *mIl-6* (IL-6, amplicon 73 bp) Fw 5′-ACAAGTCGGAGGCTTAATTACACAT, Rev 5′-TTGCCATTGCACAACTCTTTT; *mMmp9* (MMP9, amplicon 62 bp) Fw 5′-GCAGAGGCATACTTGTACCG, Rev 5′-TGCTTCTCTCCCATCATCTG; reference gene *mGapdh* (GAPDH, amplicon 124 bp) Fw 5′-CCTCGTCCCGTAGACAAAATG, Rev 5′-TGAAGGGGTCGTTGATGGC.

### 4.11. Statistical Analysis

Data were analyzed using the student’s *t*-test. All values represent means ± SEM. *p* < 0.05 (*), *p* < 0.01 (**), *p* < 0.005 (***) and *p* < 0.001 (****) were considered as statistically significant. Statistical analysis was performed using GraphPad Prism 6 software (La Jolla, CA, US).

## Supplementary Materials

Supplementary Materials can be found at http://www.mdpi.com/1422-0067/19/3/684/s1.

## Abbreviations

BID	BH3-Interacting Domain Death Agonist
DSS	Dextran Sulfate Sodium
GM-CSF	Granulocyte-Macrophage colony-Stimulating Factor
IBD	Inflammatory Bowel Disease
IL-6	Interleukin-6
LPS	Lipopolysaccharide
Q-VD-OPh	Quinoline-Val-Asp-Difluorophenoxymethylketone
TNFα	Tumor Necrosis Factor Alpha
WT	Wild-type
