# Supplementary material for: Loss of BID Delays FASL-Induced Cell Death of Mouse Neutrophils and Aggravates DSS-Induced Weight Loss
Source: Int J Mol Sci. 2018 Feb 28;19(3):684. doi: 10.3390/ijms19030684 (PMC5877545; doi:10.3390/ijms19030684)
Supplement: Supplementary file 1 [file ijms-19-00684-s001.pdf]

**Wicki S et al., Loss of BID delays FASL-induced cell death of mouse neutrophils and aggravates DSS-induced weight loss**

## ONLINE SUPPLEMENTARY INFORMATION

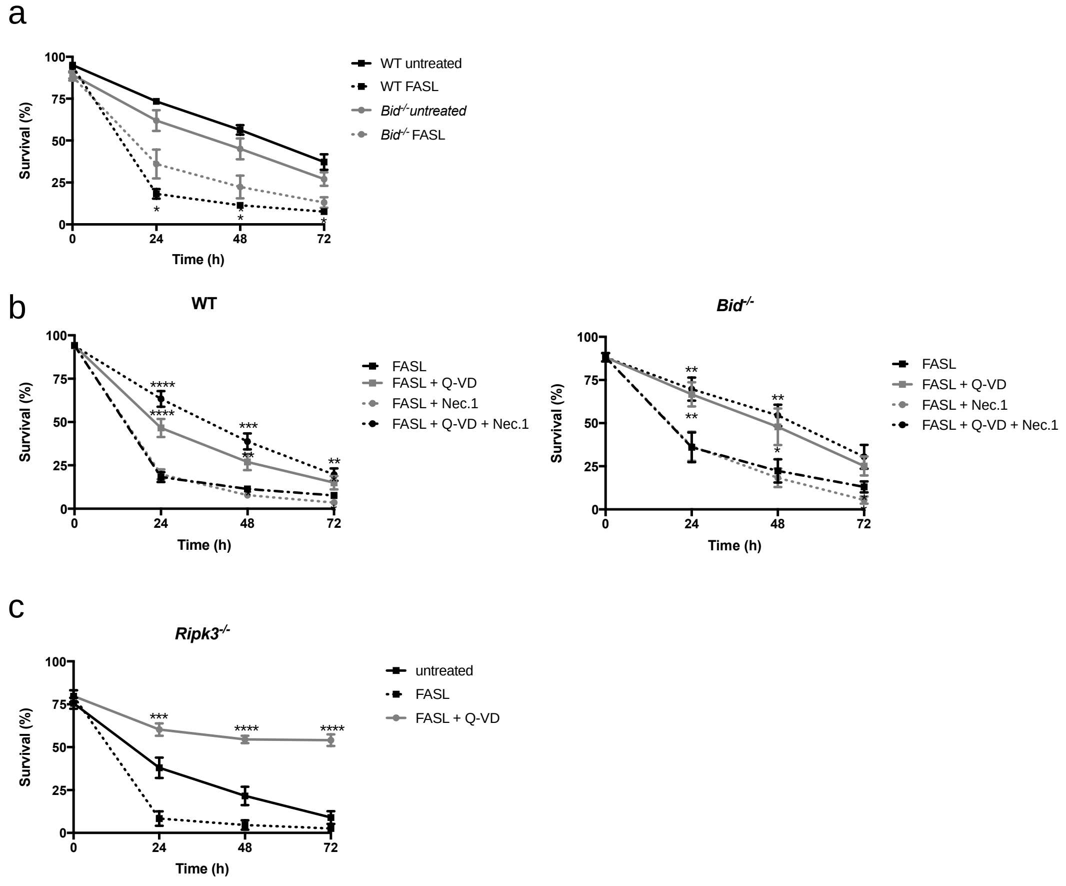


**Supplementary Figure S1 (Related to Figure 1).**

(a) WT and *Bid*$^{-/-}$ neutrophils were treated with FASL (100 ng/ml) for indicated time points. Viability was assessed by flow cytometry using GFP-Annexin V/PI exclusion. n≥4, mean ± SEM. (b) WT and *Bid*$^{-/-}$ neutrophils were pre-treated with Q-VD (20 μM) and/or Nec.1 (20 μM) for 30 min followed by administration of FASL (100 ng/ml) for indicated time points. Viability was determined by flow cytometry. n≥4, mean ± SEM. (c) *Ripk3*$^{-/-}$ neutrophils were pre-treated with Q-VD (20 μM) for 30 min prior to stimulation with FASL for indicated time points. Viability was assessed by flow cytometry. n≥4, mean ± SEM. All experiments were performed with *in vitro* differentiated neutrophils.

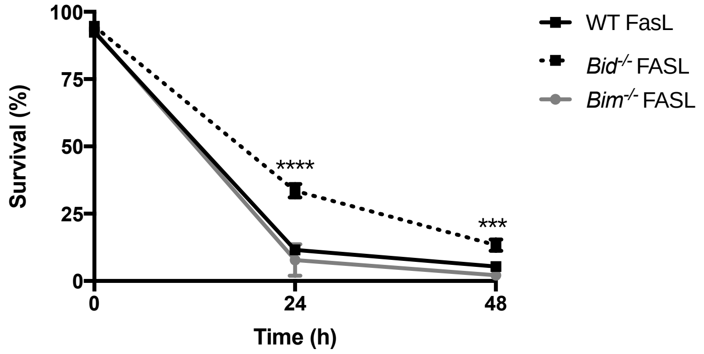


**Supplementary Figure S2 (Related to Figure 1).**

WT, *Bid*$^{-/-}$ and *Bim*$^{-/-}$ primary mouse neutrophils were treated with FASL (100 ng/ml) for indicated time points. Viability was assessed by flow cytometry using GFP-AnnexinV/ PI exclusion. n≥3, mean ± SEM.

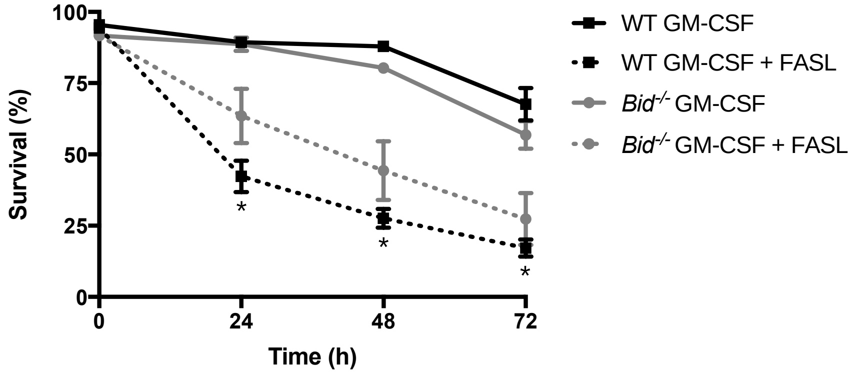


**Supplementary Figure S3 (Related to Figure 3).**

*In-vitro* differentiated WT and *Bid*$^{-/-}$ neutrophils were primed with GM-CSF (1 ng/ml) for 30 min prior to stimulation with FASL (100 ng/ml) for indicated time points. Viability was assessed by flow cytometry using GFP-Annexin V/PI exclusion. n≥3, mean ± SEM.

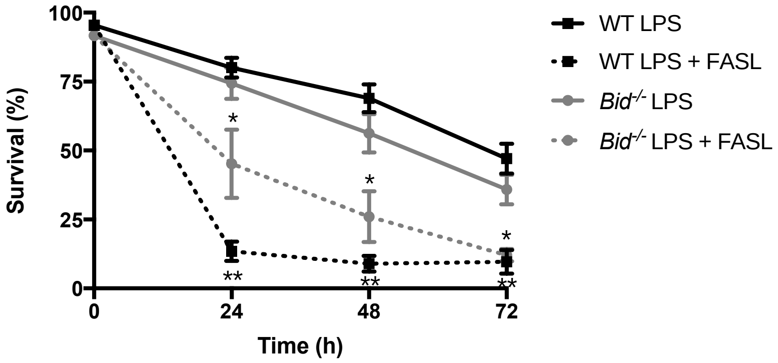


**Supplementary Figure S4 (Related to Figure 4).**

*In-vitro* differentiated WT and *Bid*$^{-/-}$ neutrophils were primed with LPS (10 ng/ml) for 30 min prior to stimulation with FASL (100 ng/ml) for indicated time points. Viability was assessed by flow cytometry using GFP-Annexin V/PI exclusion. n=3, mean ± SEM.
